# A kinome-wide RNA interference screen identifies the microtubule affinity regulating kinase MARK4 as a new regulator of primary cilium formation

**DOI:** 10.1186/2046-2530-1-S1-P30

**Published:** 2012-11-16

**Authors:** S Kuhns, K Schmidt, G Pereira

**Affiliations:** 1 DKFZ-ZMBH Alliance, German Cancer Research Center, Germany

## 

The primary cilium is a microtubule-based organelle that projects from the surface of most vertebrate cells and plays a crucial role in a number of signaling processes. Even though an increasing number of cilia-associated proteins have been identified in the past decade, the molecular mechanisms controlling primary cilia formation remain poorly understood. Here, we performed a RNA interference (RNAi) based screen using telomerase-immortalized human epithelial retina (RPE1) cells to search for kinases and kinase-associated proteins with a role in ciliogenesis. In this cell line, ciliogenesis can be induced upon serum withdrawal. In order to identify both, positive and negative regulators of ciliogenesis, we performed the RNAi screen under reduced serum conditions instead of stringent starvation. This allowed us to detect not only proteins that reduced ciliogenesis upon depletion, but also those proteins that increased the number of ciliated cells upon depletion. To screen in high-throughput, we developed an automated microscope based readout using acetylated tubulin as a cilia marker. Our primary screen identified 103 potential regulators of ciliogenesis. To validate these, we used for a secondary screen independent siRNAs and confirmed 47 positive regulators and 4 negative regulators. The confirmed hits were associated with various biological processes as intracellular signaling, cell cycle control or cytoskeleton organization. Here, we will present the in-depth characterization of the microtubule-affinity regulating kinase 4 (MARK4), which has been identified as a novel positive regulator of primary cilia assembly.

